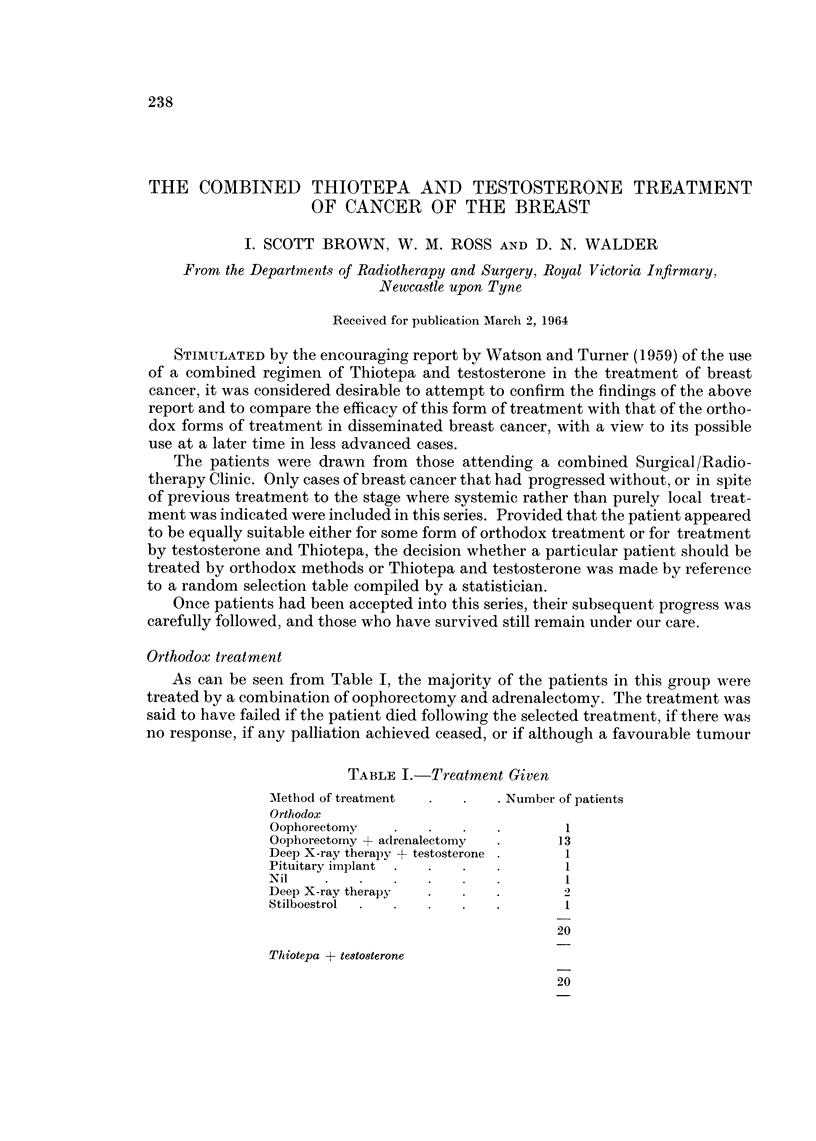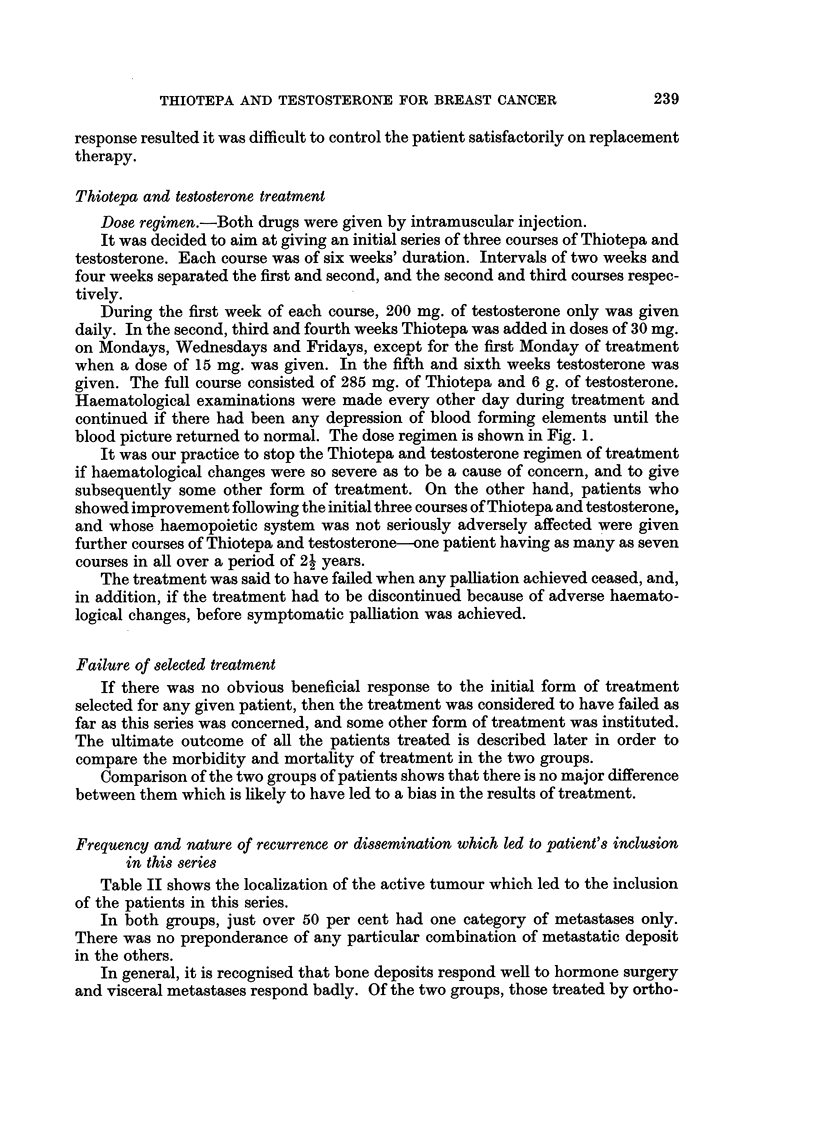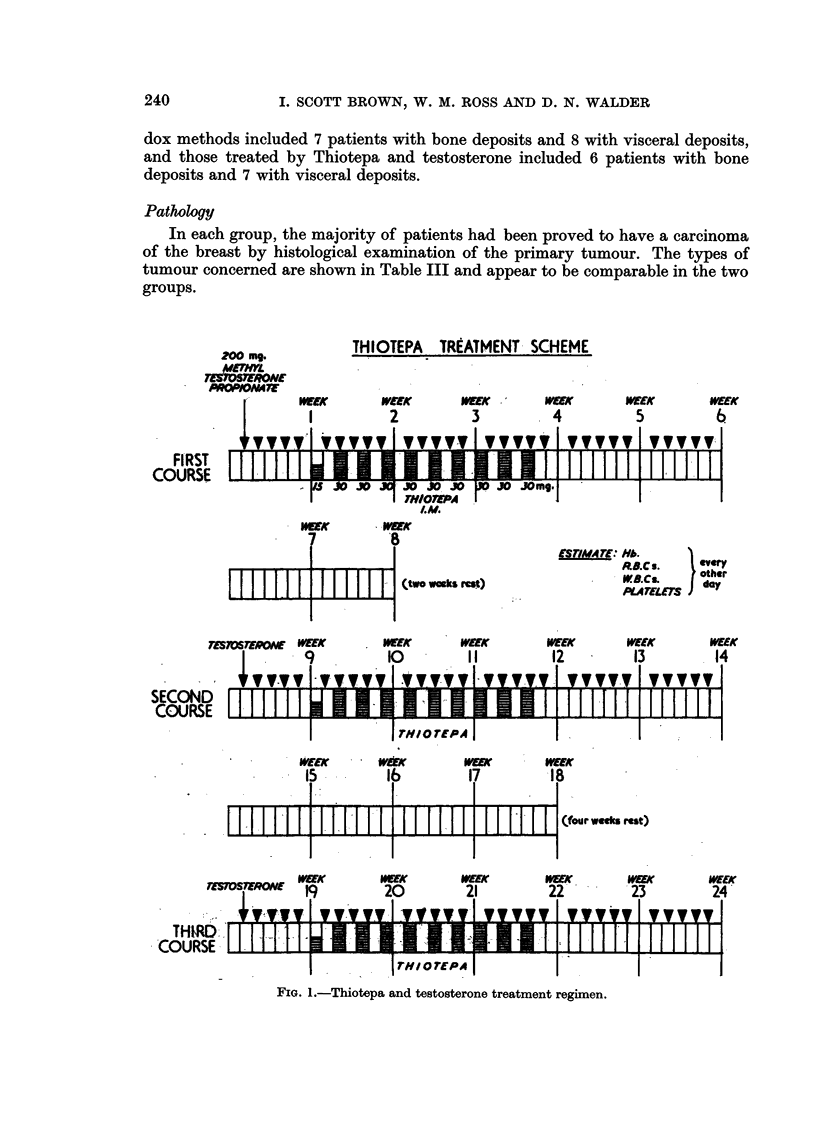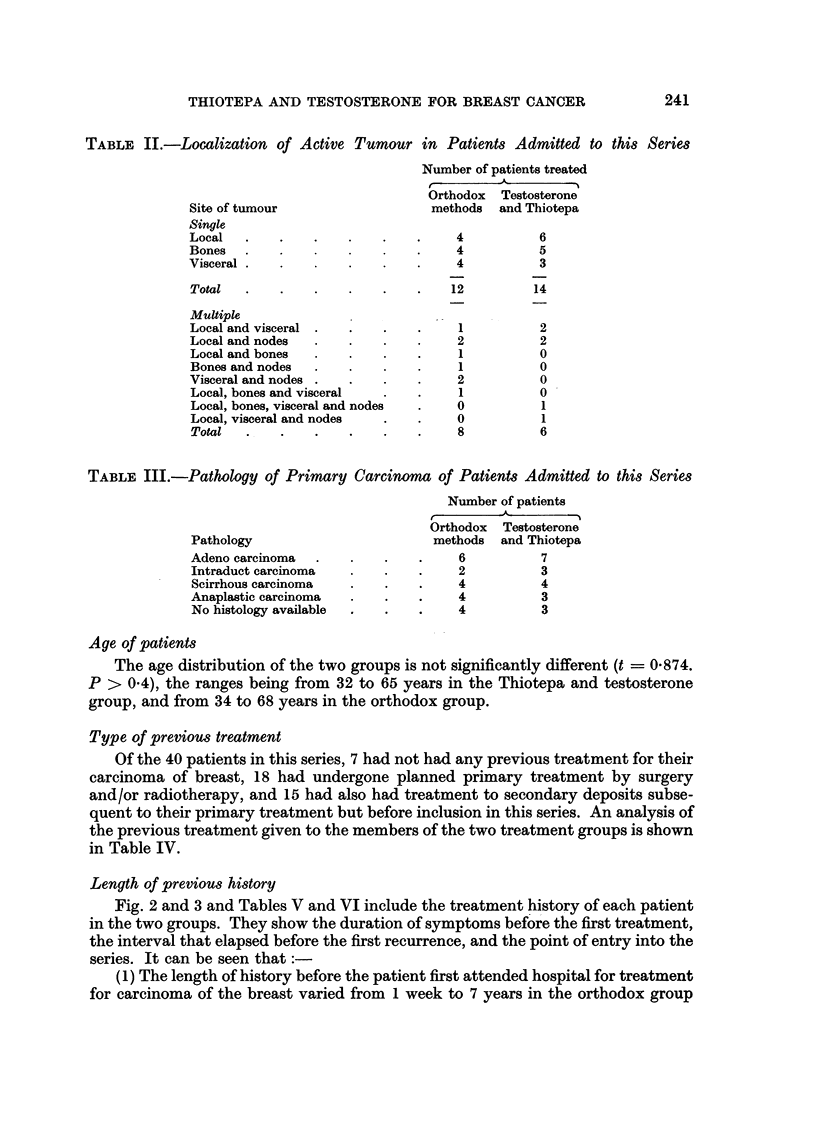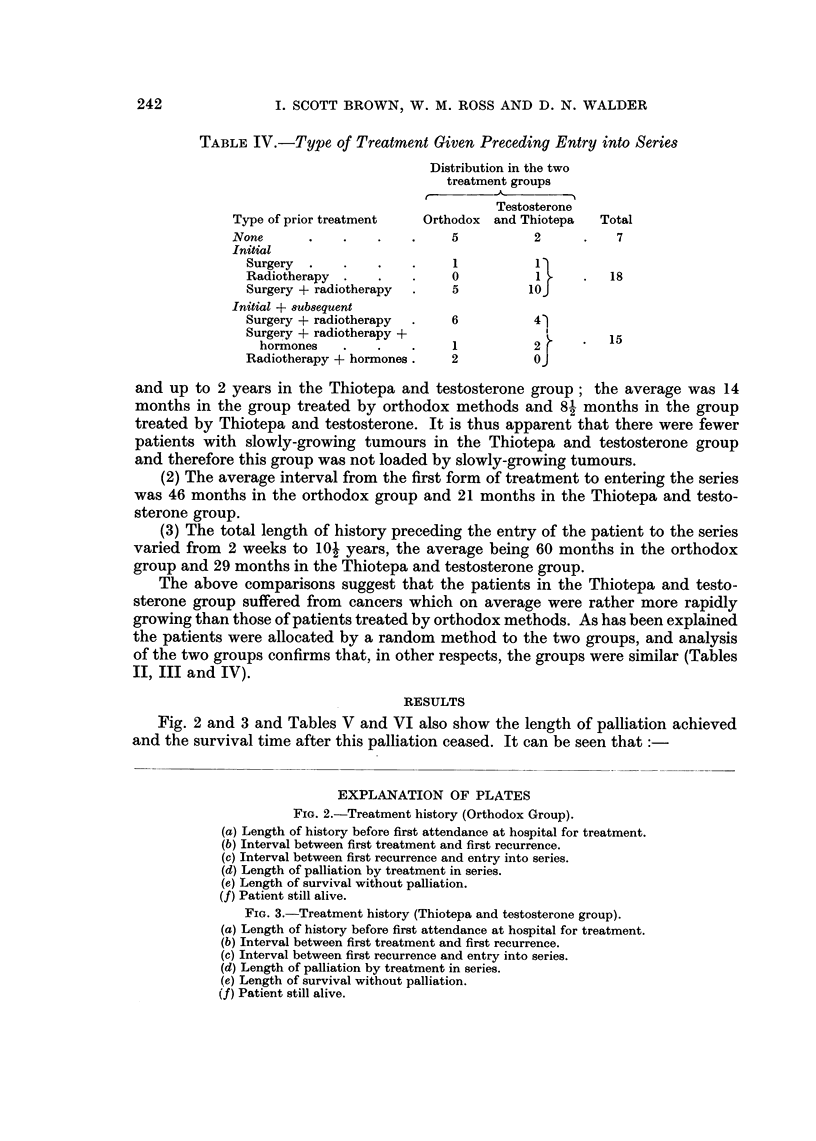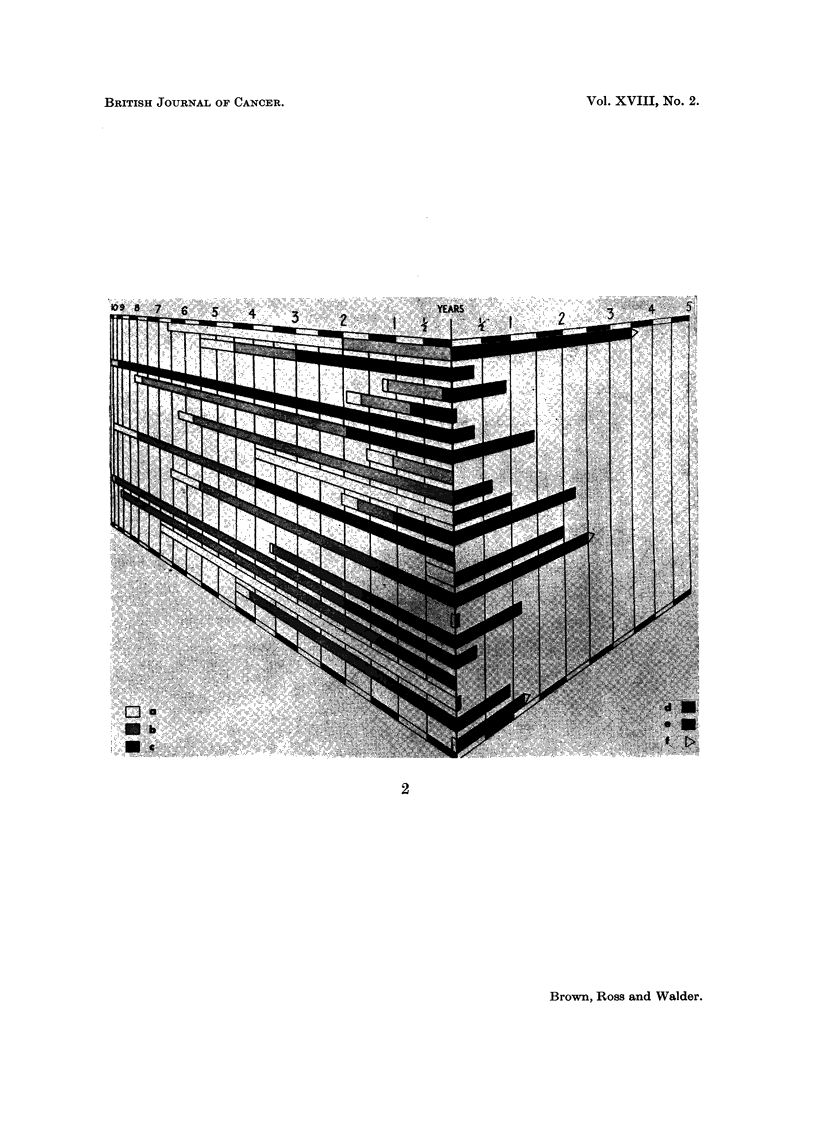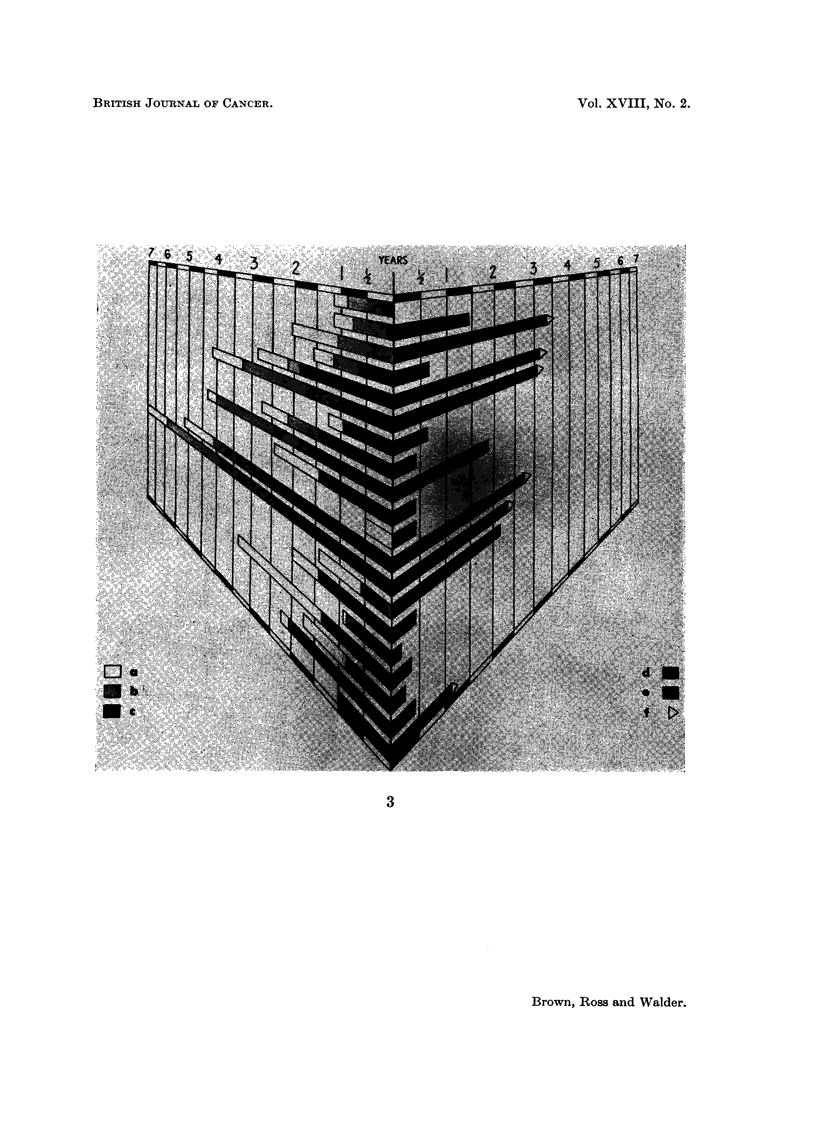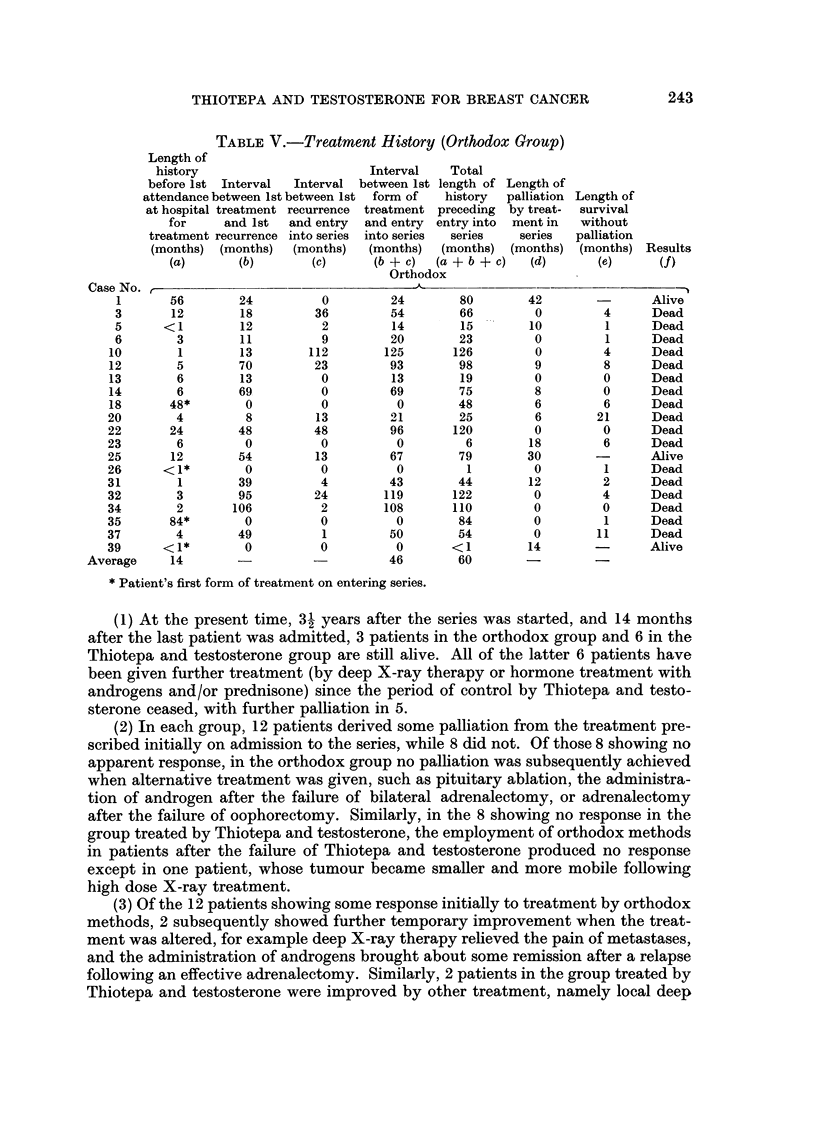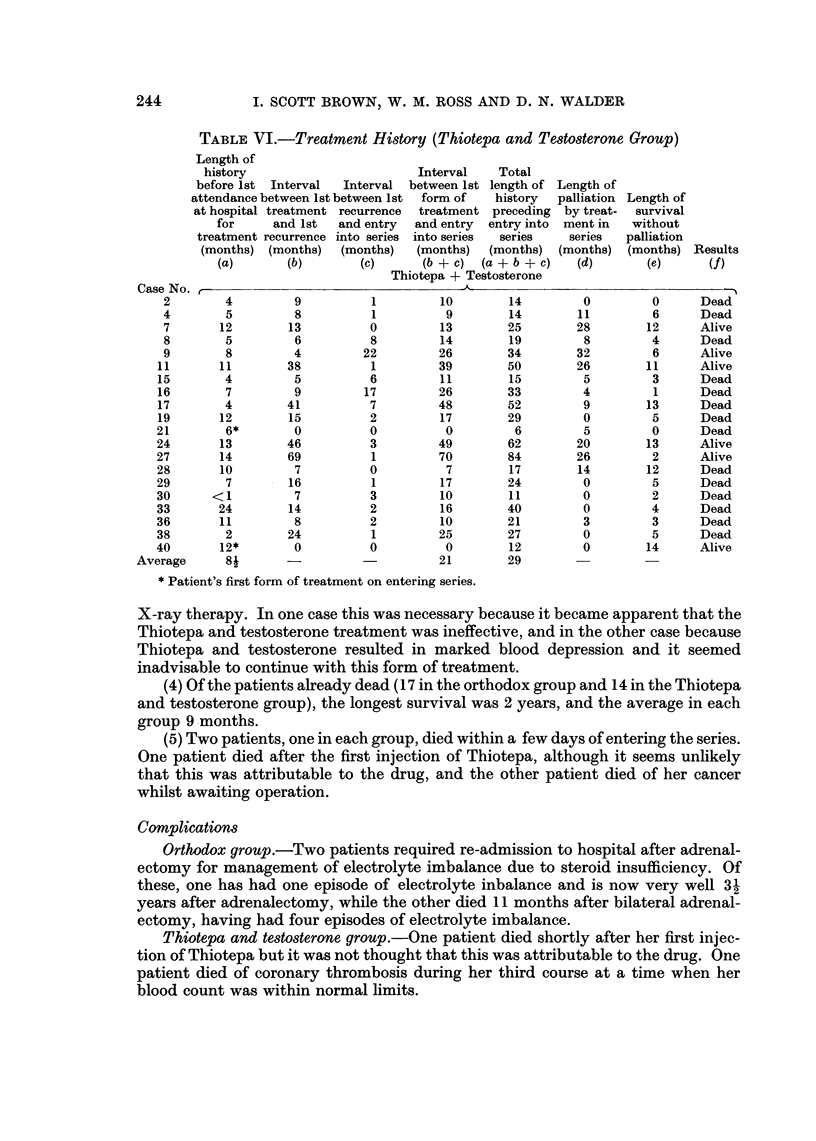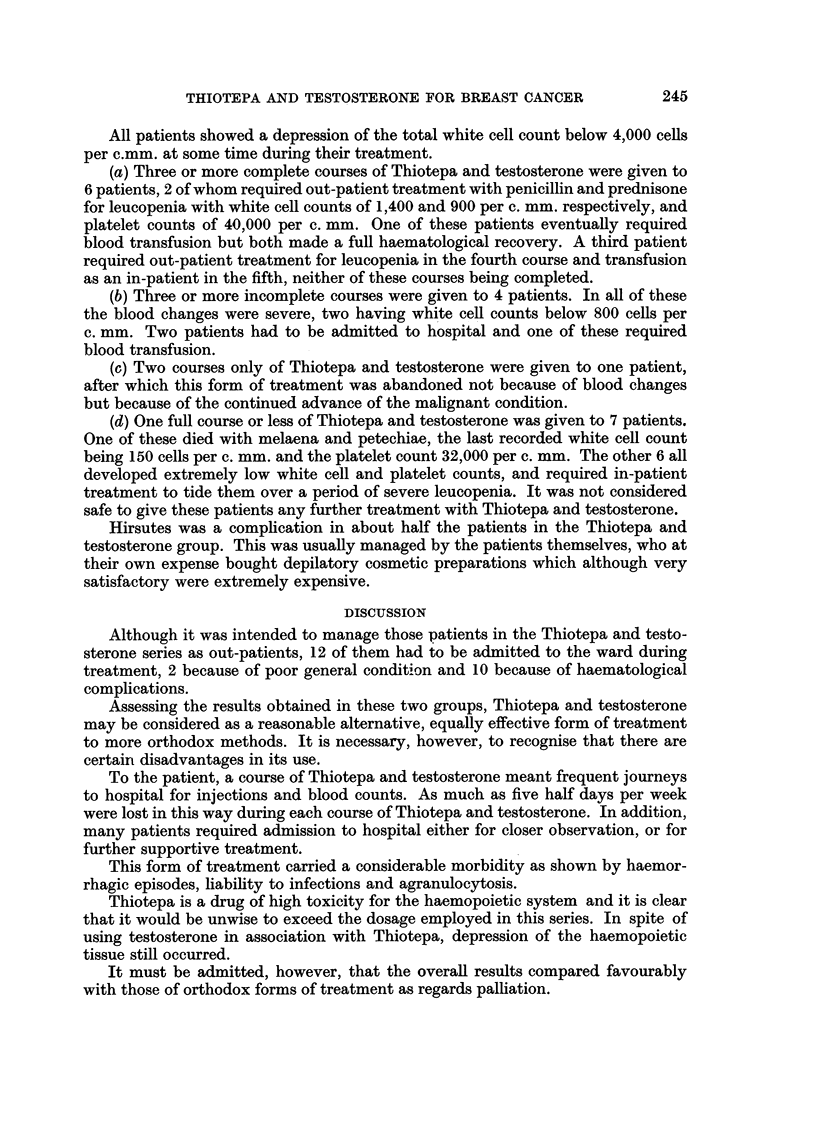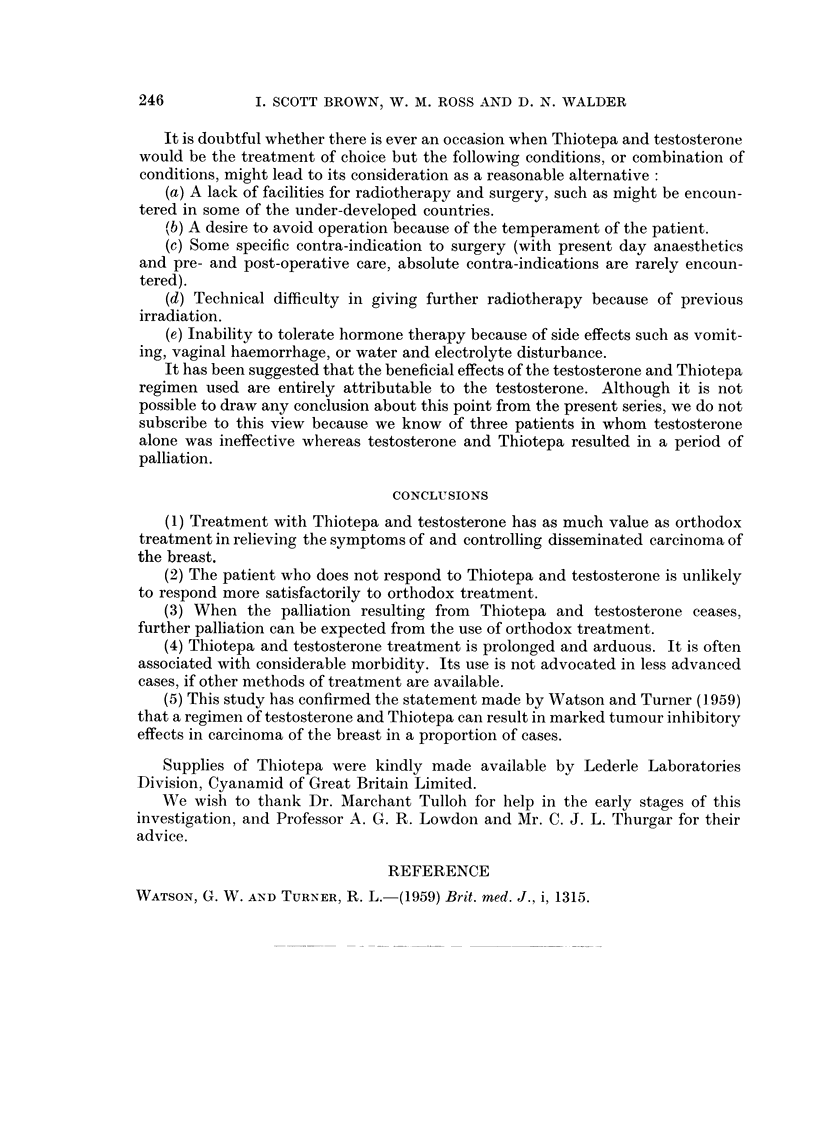# The Combined Thiotepa and Testosterone Treatment of Cancer of the Breast

**DOI:** 10.1038/bjc.1964.26

**Published:** 1964-06

**Authors:** I. Scott Brown, W. M. Ross, D. N. Walder


### THE COMBINED THIOTEPA AND TESTOSTERONE TREATMENT OF CANCER OF THE BREAST

I. SCOTT BROWN, W. M. ROSS AND D. N. WALDER

*From the Departments of Radiotherapy and Surgery, Royal Victoria Infirmary, Newcastle upon Tyne*

Received for publication March 2, 1964

STIMULATED by the encouraging report by Watson and Turner (1959) of the use of a combined regimen of Thiotepa and testosterone in the treatment of breast cancer, it was considered desirable to attempt to confirm the findings of the above report and to compare the efficacy of this form of treatment with that of the orthodox forms of treatment in disseminated breast cancer, with a view to its possible use at a later time in less advanced cases.

The patients were drawn from those attending a combined Surgical/Radiotherapy Clinic. Only cases of breast cancer that had progressed without, or in spite of previous treatment to the stage where systemic rather than purely local treatment was indicated were included in this series. Provided that the patient appeared to be equally suitable either for some form of orthodox treatment or for treatment by testosterone and Thiotepa, the decision whether a particular patient should be treated by orthodox methods or Thiotepa and testosterone was made by reference to a random selection table compiled by a statistician.

Once patients had been accepted into this series, their subsequent progress was carefully followed, and those who have survived still remain under our care.

*Orthodox treatment*

As can be seen from Table I, the majority of the patients in this group were treated by a combination of oophorectomy and adrenalectomy. The treatment was said to have failed if the patient died following the selected treatment, if there was no response, if any palliation achieved ceased, or if although a favourable tumour

TABLE I.—*Treatment Given*

| Method of treatment | Number of patients |
|---|---|
| *Orthodox* | |
| Oophorectomy | 1 |
| Oophorectomy + adrenalectomy | 13 |
| Deep X-ray therapy + testosterone | 1 |
| Pituitary implant | 1 |
| Nil | 1 |
| Deep X-ray therapy | 2 |
| Stilboestrol | 1 |
| | 20 |
| *Thiotepa + testosterone* | |
| | 20 |

response resulted it was difficult to control the patient satisfactorily on replacement therapy.

*Thiotepa and testosterone treatment*

*Dose regimen.*—Both drugs were given by intramuscular injection.

It was decided to aim at giving an initial series of three courses of Thiotepa and testosterone. Each course was of six weeks' duration. Intervals of two weeks and four weeks separated the first and second, and the second and third courses respectively.

During the first week of each course, 200 mg. of testosterone only was given daily. In the second, third and fourth weeks Thiotepa was added in doses of 30 mg. on Mondays, Wednesdays and Fridays, except for the first Monday of treatment when a dose of 15 mg. was given. In the fifth and sixth weeks testosterone was given. The full course consisted of 285 mg. of Thiotepa and 6 g. of testosterone. Haematological examinations were made every other day during treatment and continued if there had been any depression of blood forming elements until the blood picture returned to normal. The dose regimen is shown in Fig. 1.

It was our practice to stop the Thiotepa and testosterone regimen of treatment if haematological changes were so severe as to be a cause of concern, and to give subsequently some other form of treatment. On the other hand, patients who showed improvement following the initial three courses of Thiotepa and testosterone, and whose haemopoietic system was not seriously adversely affected were given further courses of Thiotepa and testosterone—one patient having as many as seven courses in all over a period of $2\frac{1}{2}$ years.

The treatment was said to have failed when any palliation achieved ceased, and, in addition, if the treatment had to be discontinued because of adverse haematological changes, before symptomatic palliation was achieved.

*Failure of selected treatment*

If there was no obvious beneficial response to the initial form of treatment selected for any given patient, then the treatment was considered to have failed as far as this series was concerned, and some other form of treatment was instituted. The ultimate outcome of all the patients treated is described later in order to compare the morbidity and mortality of treatment in the two groups.

Comparison of the two groups of patients shows that there is no major difference between them which is likely to have led to a bias in the results of treatment.

*Frequency and nature of recurrence or dissemination which led to patient's inclusion in this series*

Table II shows the localization of the active tumour which led to the inclusion of the patients in this series.

In both groups, just over 50 per cent had one category of metastases only. There was no preponderance of any particular combination of metastatic deposit in the others.

In general, it is recognised that bone deposits respond well to hormone surgery and visceral metastases respond badly. Of the two groups, those treated by orthodox methods included 7 patients with bone deposits and 8 with visceral deposits, and those treated by Thiotepa and testosterone included 6 patients with bone deposits and 7 with visceral deposits.

*Pathology*

In each group, the majority of patients had been proved to have a carcinoma of the breast by histological examination of the primary tumour. The types of tumour concerned are shown in Table III and appear to be comparable in the two groups.

FIG. 1.—Thiotepa and testosterone treatment regimen.

TABLE II.—*Localization of Active Tumour in Patients Admitted to this Series*

| Site of tumour | Number of patients treated: Orthodox methods | Testosterone and Thiotepa |
|---|---|---|
| *Single* | | |
| Local | 4 | 6 |
| Bones | 4 | 5 |
| Visceral | 4 | 3 |
| *Total* | 12 | 14 |
| *Multiple* | | |
| Local and visceral | 1 | 2 |
| Local and nodes | 2 | 2 |
| Local and bones | 1 | 0 |
| Bones and nodes | 1 | 0 |
| Visceral and nodes | 2 | 0 |
| Local, bones and visceral | 1 | 0 |
| Local, bones, visceral and nodes | 0 | 1 |
| Local, visceral and nodes | 0 | 1 |
| *Total* | 8 | 6 |

TABLE III.—*Pathology of Primary Carcinoma of Patients Admitted to this Series*

| Pathology | Number of patients: Orthodox methods | Testosterone and Thiotepa |
|---|---|---|
| Adeno carcinoma | 6 | 7 |
| Intraduct carcinoma | 2 | 3 |
| Scirrhous carcinoma | 4 | 4 |
| Anaplastic carcinoma | 4 | 3 |
| No histology available | 4 | 3 |

*Age of patients*

The age distribution of the two groups is not significantly different ($t = 0{\cdot}874$. $P > 0{\cdot}4$), the ranges being from 32 to 65 years in the Thiotepa and testosterone group, and from 34 to 68 years in the orthodox group.

*Type of previous treatment*

Of the 40 patients in this series, 7 had not had any previous treatment for their carcinoma of breast, 18 had undergone planned primary treatment by surgery and/or radiotherapy, and 15 had also had treatment to secondary deposits subsequent to their primary treatment but before inclusion in this series. An analysis of the previous treatment given to the members of the two treatment groups is shown in Table IV.

*Length of previous history*

Fig. 2 and 3 and Tables V and VI include the treatment history of each patient in the two groups. They show the duration of symptoms before the first treatment, the interval that elapsed before the first recurrence, and the point of entry into the series. It can be seen that :—

(1) The length of history before the patient first attended hospital for treatment for carcinoma of the breast varied from 1 week to 7 years in the orthodox group

TABLE IV.—*Type of Treatment Given Preceding Entry into Series*

| Type of prior treatment | Distribution in the two treatment groups: Orthodox | Testosterone and Thiotepa | Total |
|---|---|---|---|
| *None* | 5 | 2 | 7 |
| *Initial* | | | |
| Surgery | 1 | 1 | 18 |
| Radiotherapy | 0 | 1 | |
| Surgery + radiotherapy | 5 | 10 | |
| *Initial + subsequent* | | | |
| Surgery + radiotherapy | 6 | 4 | 15 |
| Surgery + radiotherapy + hormones | 1 | 2 | |
| Radiotherapy + hormones | 2 | 0 | |

and up to 2 years in the Thiotepa and testosterone group; the average was 14 months in the group treated by orthodox methods and 8½ months in the group treated by Thiotepa and testosterone. It is thus apparent that there were fewer patients with slowly-growing tumours in the Thiotepa and testosterone group and therefore this group was not loaded by slowly-growing tumours.

(2) The average interval from the first form of treatment to entering the series was 46 months in the orthodox group and 21 months in the Thiotepa and testosterone group.

(3) The total length of history preceding the entry of the patient to the series varied from 2 weeks to 10½ years, the average being 60 months in the orthodox group and 29 months in the Thiotepa and testosterone group.

The above comparisons suggest that the patients in the Thiotepa and testosterone group suffered from cancers which on average were rather more rapidly growing than those of patients treated by orthodox methods. As has been explained the patients were allocated by a random method to the two groups, and analysis of the two groups confirms that, in other respects, the groups were similar (Tables II, III and IV).

### RESULTS

Fig. 2 and 3 and Tables V and VI also show the length of palliation achieved and the survival time after this palliation ceased. It can be seen that :—

---

### EXPLANATION OF PLATES

FIG. 2.—Treatment history (Orthodox Group).

(*a*) Length of history before first attendance at hospital for treatment.
(*b*) Interval between first treatment and first recurrence.
(*c*) Interval between first recurrence and entry into series.
(*d*) Length of palliation by treatment in series.
(*e*) Length of survival without palliation.
(*f*) Patient still alive.

FIG. 3.—Treatment history (Thiotepa and testosterone group).

(*a*) Length of history before first attendance at hospital for treatment.
(*b*) Interval between first treatment and first recurrence.
(*c*) Interval between first recurrence and entry into series.
(*d*) Length of palliation by treatment in series.
(*e*) Length of survival without palliation.
(*f*) Patient still alive.

2

Brown, Ross and Walder.

3

Brown, Ross and Walder.

TABLE V.—*Treatment History* (*Orthodox Group*)

| Case No. | Length of history before 1st attendance at hospital for treatment (months) (*a*) | Interval between 1st treatment and 1st recurrence (months) (*b*) | Interval between 1st recurrence and entry into series (months) (*c*) | Interval between 1st form of treatment and entry into series (months) (*b* + *c*) | Total length of history preceding entry into series (months) (*a* + *b* + *c*) | Length of palliation by treatment in series (months) (*d*) | Length of survival without palliation (months) (*e*) | Results (*f*) |
|---|---|---|---|---|---|---|---|---|
| | Orthodox | | | | | | | |
| 1 | 56 | 24 | 0 | 24 | 80 | 42 | — | Alive |
| 3 | 12 | 18 | 36 | 54 | 66 | 0 | 4 | Dead |
| 5 | <1 | 12 | 2 | 14 | 15 | 10 | 1 | Dead |
| 6 | 3 | 11 | 9 | 20 | 23 | 0 | 1 | Dead |
| 10 | 1 | 13 | 112 | 125 | 126 | 0 | 4 | Dead |
| 12 | 5 | 70 | 23 | 93 | 98 | 9 | 8 | Dead |
| 13 | 6 | 13 | 0 | 13 | 19 | 0 | 0 | Dead |
| 14 | 6 | 69 | 0 | 69 | 75 | 8 | 0 | Dead |
| 18 | 48* | 0 | 0 | 0 | 48 | 6 | 6 | Dead |
| 20 | 4 | 8 | 13 | 21 | 25 | 6 | 21 | Dead |
| 22 | 24 | 48 | 48 | 96 | 120 | 0 | 0 | Dead |
| 23 | 6 | 0 | 0 | 0 | 6 | 18 | 6 | Dead |
| 25 | 12 | 54 | 13 | 67 | 79 | 30 | — | Alive |
| 26 | <1* | 0 | 0 | 0 | 1 | 0 | 1 | Dead |
| 31 | 1 | 39 | 4 | 43 | 44 | 12 | 2 | Dead |
| 32 | 3 | 95 | 24 | 119 | 122 | 0 | 4 | Dead |
| 34 | 2 | 106 | 2 | 108 | 110 | 0 | 0 | Dead |
| 35 | 84* | 0 | 0 | 0 | 84 | 0 | 1 | Dead |
| 37 | 4 | 49 | 1 | 50 | 54 | 0 | 11 | Dead |
| 39 | <1* | 0 | 0 | 0 | <1 | 14 | — | Alive |
| Average | 14 | — | — | 46 | 60 | — | — | |

* Patient's first form of treatment on entering series.

(1) At the present time, $3\frac{1}{2}$ years after the series was started, and 14 months after the last patient was admitted, 3 patients in the orthodox group and 6 in the Thiotepa and testosterone group are still alive. All of the latter 6 patients have been given further treatment (by deep X-ray therapy or hormone treatment with androgens and/or prednisone) since the period of control by Thiotepa and testosterone ceased, with further palliation in 5.

(2) In each group, 12 patients derived some palliation from the treatment prescribed initially on admission to the series, while 8 did not. Of those 8 showing no apparent response, in the orthodox group no palliation was subsequently achieved when alternative treatment was given, such as pituitary ablation, the administration of androgen after the failure of bilateral adrenalectomy, or adrenalectomy after the failure of oophorectomy. Similarly, in the 8 showing no response in the group treated by Thiotepa and testosterone, the employment of orthodox methods in patients after the failure of Thiotepa and testosterone produced no response except in one patient, whose tumour became smaller and more mobile following high dose X-ray treatment.

(3) Of the 12 patients showing some response initially to treatment by orthodox methods, 2 subsequently showed further temporary improvement when the treatment was altered, for example deep X-ray therapy relieved the pain of metastases, and the administration of androgens brought about some remission after a relapse following an effective adrenalectomy. Similarly, 2 patients in the group treated by Thiotepa and testosterone were improved by other treatment, namely local deep

TABLE VI.—*Treatment History (Thiotepa and Testosterone Group)*

| Case No. | Length of history before 1st attendance at hospital for treatment (months) (*a*) | Interval between 1st treatment and 1st recurrence (months) (*b*) | Interval between 1st recurrence and entry into series (months) (*c*) | Interval between 1st form of treatment and entry into series (months) (*b* + *c*) | Total length of history preceding entry into series (months) (*a* + *b* + *c*) | Length of palliation by treatment in series (months) (*d*) | Length of survival without palliation (months) (*e*) | Results (*f*) |
|---|---|---|---|---|---|---|---|---|
| | Thiotepa + Testosterone | | | | | | | |
| 2 | 4 | 9 | 1 | 10 | 14 | 0 | 0 | Dead |
| 4 | 5 | 8 | 1 | 9 | 14 | 11 | 6 | Dead |
| 7 | 12 | 13 | 0 | 13 | 25 | 28 | 12 | Alive |
| 8 | 5 | 6 | 8 | 14 | 19 | 8 | 4 | Dead |
| 9 | 8 | 4 | 22 | 26 | 34 | 32 | 6 | Alive |
| 11 | 11 | 38 | 1 | 39 | 50 | 26 | 11 | Alive |
| 15 | 4 | 5 | 6 | 11 | 15 | 5 | 3 | Dead |
| 16 | 7 | 9 | 17 | 26 | 33 | 4 | 1 | Dead |
| 17 | 4 | 41 | 7 | 48 | 52 | 9 | 13 | Dead |
| 19 | 12 | 15 | 2 | 17 | 29 | 0 | 5 | Dead |
| 21 | 6* | 0 | 0 | 0 | 6 | 5 | 0 | Dead |
| 24 | 13 | 46 | 3 | 49 | 62 | 20 | 13 | Alive |
| 27 | 14 | 69 | 1 | 70 | 84 | 26 | 2 | Alive |
| 28 | 10 | 7 | 0 | 7 | 17 | 14 | 12 | Dead |
| 29 | 7 | 16 | 1 | 17 | 24 | 0 | 5 | Dead |
| 30 | $<1$ | 7 | 3 | 10 | 11 | 0 | 2 | Dead |
| 33 | 24 | 14 | 2 | 16 | 40 | 0 | 4 | Dead |
| 36 | 11 | 8 | 2 | 10 | 21 | 3 | 3 | Dead |
| 38 | 2 | 24 | 1 | 25 | 27 | 0 | 5 | Dead |
| 40 | 12* | 0 | 0 | 0 | 12 | 0 | 14 | Alive |
| Average | $8\frac{1}{2}$ | — | — | 21 | 29 | — | — | |

* Patient's first form of treatment on entering series.

X-ray therapy. In one case this was necessary because it became apparent that the Thiotepa and testosterone treatment was ineffective, and in the other case because Thiotepa and testosterone resulted in marked blood depression and it seemed inadvisable to continue with this form of treatment.

(4) Of the patients already dead (17 in the orthodox group and 14 in the Thiotepa and testosterone group), the longest survival was 2 years, and the average in each group 9 months.

(5) Two patients, one in each group, died within a few days of entering the series. One patient died after the first injection of Thiotepa, although it seems unlikely that this was attributable to the drug, and the other patient died of her cancer whilst awaiting operation.

*Complications*

*Orthodox group.*—Two patients required re-admission to hospital after adrenalectomy for management of electrolyte imbalance due to steroid insufficiency. Of these, one has had one episode of electrolyte inbalance and is now very well $3\frac{1}{2}$ years after adrenalectomy, while the other died 11 months after bilateral adrenalectomy, having had four episodes of electrolyte imbalance.

*Thiotepa and testosterone group.*—One patient died shortly after her first injection of Thiotepa but it was not thought that this was attributable to the drug. One patient died of coronary thrombosis during her third course at a time when her blood count was within normal limits.

All patients showed a depression of the total white cell count below 4,000 cells per c.mm. at some time during their treatment.

(*a*) Three or more complete courses of Thiotepa and testosterone were given to 6 patients, 2 of whom required out-patient treatment with penicillin and prednisone for leucopenia with white cell counts of 1,400 and 900 per c. mm. respectively, and platelet counts of 40,000 per c. mm. One of these patients eventually required blood transfusion but both made a full haematological recovery. A third patient required out-patient treatment for leucopenia in the fourth course and transfusion as an in-patient in the fifth, neither of these courses being completed.

(*b*) Three or more incomplete courses were given to 4 patients. In all of these the blood changes were severe, two having white cell counts below 800 cells per c. mm. Two patients had to be admitted to hospital and one of these required blood transfusion.

(*c*) Two courses only of Thiotepa and testosterone were given to one patient, after which this form of treatment was abandoned not because of blood changes but because of the continued advance of the malignant condition.

(*d*) One full course or less of Thiotepa and testosterone was given to 7 patients. One of these died with melaena and petechiae, the last recorded white cell count being 150 cells per c. mm. and the platelet count 32,000 per c. mm. The other 6 all developed extremely low white cell and platelet counts, and required in-patient treatment to tide them over a period of severe leucopenia. It was not considered safe to give these patients any further treatment with Thiotepa and testosterone.

Hirsutes was a complication in about half the patients in the Thiotepa and testosterone group. This was usually managed by the patients themselves, who at their own expense bought depilatory cosmetic preparations which although very satisfactory were extremely expensive.

### DISCUSSION

Although it was intended to manage those patients in the Thiotepa and testosterone series as out-patients, 12 of them had to be admitted to the ward during treatment, 2 because of poor general condition and 10 because of haematological complications.

Assessing the results obtained in these two groups, Thiotepa and testosterone may be considered as a reasonable alternative, equally effective form of treatment to more orthodox methods. It is necessary, however, to recognise that there are certain disadvantages in its use.

To the patient, a course of Thiotepa and testosterone meant frequent journeys to hospital for injections and blood counts. As much as five half days per week were lost in this way during each course of Thiotepa and testosterone. In addition, many patients required admission to hospital either for closer observation, or for further supportive treatment.

This form of treatment carried a considerable morbidity as shown by haemorrhagic episodes, liability to infections and agranulocytosis.

Thiotepa is a drug of high toxicity for the haemopoietic system and it is clear that it would be unwise to exceed the dosage employed in this series. In spite of using testosterone in association with Thiotepa, depression of the haemopoietic tissue still occurred.

It must be admitted, however, that the overall results compared favourably with those of orthodox forms of treatment as regards palliation.

It is doubtful whether there is ever an occasion when Thiotepa and testosterone would be the treatment of choice but the following conditions, or combination of conditions, might lead to its consideration as a reasonable alternative :

(*a*) A lack of facilities for radiotherapy and surgery, such as might be encountered in some of the under-developed countries.

(*b*) A desire to avoid operation because of the temperament of the patient.

(*c*) Some specific contra-indication to surgery (with present day anaesthetics and pre- and post-operative care, absolute contra-indications are rarely encountered).

(*d*) Technical difficulty in giving further radiotherapy because of previous irradiation.

(*e*) Inability to tolerate hormone therapy because of side effects such as vomiting, vaginal haemorrhage, or water and electrolyte disturbance.

It has been suggested that the beneficial effects of the testosterone and Thiotepa regimen used are entirely attributable to the testosterone. Although it is not possible to draw any conclusion about this point from the present series, we do not subscribe to this view because we know of three patients in whom testosterone alone was ineffective whereas testosterone and Thiotepa resulted in a period of palliation.

### CONCLUSIONS

(1) Treatment with Thiotepa and testosterone has as much value as orthodox treatment in relieving the symptoms of and controlling disseminated carcinoma of the breast.

(2) The patient who does not respond to Thiotepa and testosterone is unlikely to respond more satisfactorily to orthodox treatment.

(3) When the palliation resulting from Thiotepa and testosterone ceases, further palliation can be expected from the use of orthodox treatment.

(4) Thiotepa and testosterone treatment is prolonged and arduous. It is often associated with considerable morbidity. Its use is not advocated in less advanced cases, if other methods of treatment are available.

(5) This study has confirmed the statement made by Watson and Turner (1959) that a regimen of testosterone and Thiotepa can result in marked tumour inhibitory effects in carcinoma of the breast in a proportion of cases.

Supplies of Thiotepa were kindly made available by Lederle Laboratories Division, Cyanamid of Great Britain Limited.

We wish to thank Dr. Marchant Tulloh for help in the early stages of this investigation, and Professor A. G. R. Lowdon and Mr. C. J. L. Thurgar for their advice.

### REFERENCE

WATSON, G. W. AND TURNER, R. L.—(1959) *Brit. med. J.*, i, 1315.